# Spermidine Enhances Heat Tolerance of Rice Seeds by Modulating Endogenous Starch and Polyamine Metabolism

**DOI:** 10.3390/molecules24071395

**Published:** 2019-04-09

**Authors:** Yuying Fu, Qingqing Gu, Qian Dong, Zhihao Zhang, Cheng Lin, Weimin Hu, Ronghui Pan, Yajing Guan, Jin Hu

**Affiliations:** Seed Science Center, College of Agriculture and Biotechnology, Zhejiang University, Hangzhou 310029, China; yyfu1991@sina.com (Y.F.); newbisile@163.com (Q.G.); dongqian777@zju.edu.cn (Q.D.); 21716034@zju.edu.cn (Z.Z.); lincheng837243270@163.com (C.L.); huwm168@zju.edu.cn (W.H.); panr@zju.edu.cn (R.P.); jhu@zju.edu.cn (J.H.)

**Keywords:** spermidine, high temperature, grain filling, starch, polyamines

## Abstract

Polyamines have been reported to be involved in grain filling and they might contribute to the construction of heat resistance of some cereals. In this study, the hybrid rice ‘YLY 689’ was used to explore the possible effects of exogenous spermidine (Spd) on seed quality under high temperature during the filling stage. Rice spikes were treated with Spd or its synthesis inhibitor cyclohexylamine (CHA) after pollination, and then the rice plants were transferred to 40 °C for 5-day heat treatment. The results showed that, compared with the control under high temperature, Spd pretreatment significantly improved the germination percentage, germination index, vigor index, seedling shoot height, and dry weight of seeds harvested at 35 days after pollination, while the CHA significantly decreased the seed germination and seedling growth. Meanwhile, Spd significantly increased the peroxidase (POD) activity and decreased the malondialdehyde (MDA) content in seeds. In addition, after spraying with Spd, the endogenous content of spermidine and spermine and the expression of their synthetic genes, *spermidine synthase* (*SPDSYN)* and *spermine synthase* (*SPMS1* and *SPMS2*), significantly increased, whereas the accumulation of amylose and total starch and the expression of their related synthase genes, *soluble starch synthase II-3* (*SS II-3*) and *granules bound starch synthase* *I* (*GBSSI*), also increased to some extent. The data suggests that exogenous Spd pretreatment could alleviate the negative impacts of high temperature stress on rice seed grain filling and improve the rice seed quality to some extent, which might be partly caused by up-regulating endogenous polyamines and starch metabolism.

## 1. Introduction

Most abiotic stressors, including heat, cold, drought, and salinity, have easily led to crop yield and quality decline [1,2]. At present, as a result of the increase in global temperature, the risk of crops suffering from high temperature stress has also increased [3]. Rice (*Oryza sativa* L.) is one of the most important food crops in the world and provides staple food for more than half of the world’s population [4]. However, if high temperatures occur during the seed development, especially at the grain-filling stage, the amylose content of rice seeds is reduced, changing the fine amylopectin structure and producing more chalky grains [5,6]. This also reduces the rice yield and seed quality [7,8]. The occurrence of chalky grains of rice is typically caused by the unusual expressions of genes encoding starch synthase enzymes [9,10].

Polyamines (PAs), mainly including putrescine (Put), spermidine (Spd), and spermine (Spm), are involved in various processes of plant growth and development, such as seed development [11], seed germination [12], seed dormancy [13], and seedling growth [14]. Huang et al. (2017) found that soaking seeds in Spd significantly enhanced seed vigor, while exogenous cyclohexylamine (CHA) significantly inhibited seed germination and reduced seed vigor [15]. PAs were also related to plant stress resistance [16,17,18]. Mostofa et al. (2014) found that foliar spray with 1 mM Spd enhanced the antioxidative and glyoxalase systems and reduced malondialdehyde (MDA) content in 14-day-old healthy rice seedlings in a 42 °C stress treatment [19]. Zhang et al. (2017) found that the application of Spd enhanced the heat tolerance of tall fescue by protecting cell membrane stability, increasing antioxidant enzymes activity, and stabilizing the structure of nucleic acids in leaves under 44 °C [20]. As the above-mentioned studies have indicated, Spd is involved in heat tolerance in higher plants, which might act by protecting membrane stability and enhancing the reactive oxygen species (ROS) scavenging system.

In addition, Spd was proposed to be involved in rice grain filling. Chen et al. found that the exogenous application of Spd significantly increased Spd and Spm content, grain-filling rate, and grain weight in rice spikelets [21]. Cao et al. (2016) found that the content of Spd and Spm in superior grains of rice was significantly higher than that in inferior grains [22]. The content of Spd and Spm in grains was positively correlated with grain plumpness in the grain filling stage. Saleethong et al. (2013) demonstrated that 1 mM Spd treatment at the early booting stage alleviated the adverse effects of NaCl stress during the grain filling period, leading to an improvement in rice yield [23]. The content of endogenous PAs in the heat-resistant rice lines was more stable than that in the heat-sensitive lines under high temperature stress [23]. While polyamines are related to rice grain filling and general plant heat tolerance, it is unknown how Spd regulates rice grain filling under high temperature stress during seed development.

In China, agricultural loss caused by heat damage in hybrid rice is more serious than that in conventional rice fields [24]. However, there are few reports focused on the heat resistance of hybrid rice, especially on the role of Spd in the high temperature tolerance of hybrid rice. Therefore, in this study, we analyzed the effects of exogenous Spd on grain filling during seed development and seed quality of hybrid rice ‘YLY 689’ under high temperature stress. Our results showed that Spd pretreatment obviously increased the seed thickness, 1000-grain weight, and seed vigor under high temperature. Exogenous Spd significantly increased the endogenous Spd and Spm content and up-regulated expressions of *SPDS* and *SPMS*. It also up-regulated the expressions of starch synthetases genes (*SS II-3*, *GBSSI*), and led to the increased accumulation of amylose in seeds. The results demonstrated that exogenous Spd enhanced the seed size and quality under high temperature stress, most likely by modulating endogenous starch and polyamine metabolism in rice.

## 2. Results

### 2.1. Effects of Exogenous Spermidine on Rice Seed Quality under High Temperature Stress during the Early Developmental Stage of Seeds

**Figure 1:**
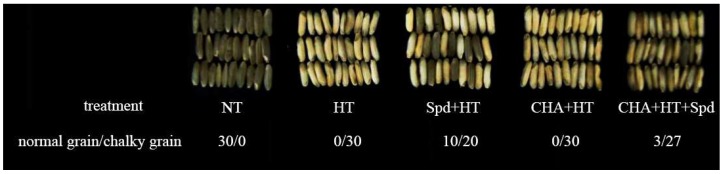
Effect of exogenous spermidine (Spd) and cyclohexylamine (CHA) on the ratio of chalky grains to normal grains of rice under high-temperature stress. NT: Normal temperature control; HT: High temperature control; Spd + HT: 1.5 mM exogenous Spd treatment + high temperature; CHA+HT: 20 mM exogenous CHA treatment + high temperature; CHA + HT + Spd: 20 mM exogenous CHA treatment + high temperature + exogenous 1.5 mM Spd.

After high temperature treatment (HT) during the early development of rice seed, the ratio of chalky seeds to normal seeds increased significantly compared with the normal temperature treatment (NT) (Figure 1). However, this was ameliorated by exogenous Spd pretreatment (Spd + HT). The cyclohexylamine (CHA) (CHA + HT) showed no significant difference with HT as the chalky seeds of HT reached 100%. However, the ratio decreased after spraying with additional Spd (CHA + HT + Spd) (Figure 1).

**Figure 2:**
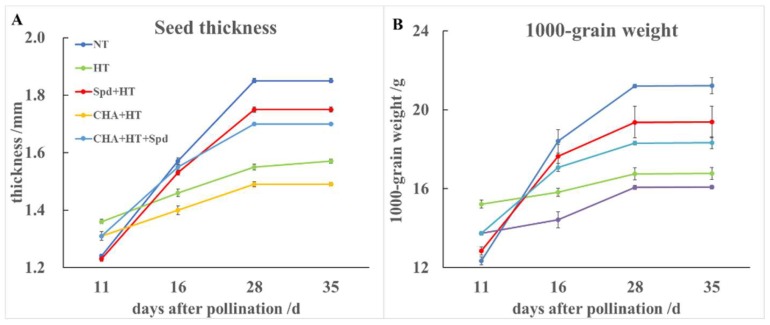
Effects of exogenous Spd and CHA on (**A**) the thickness and (**B**) the 1000-grain weight of rice seeds at different developmental stages under high-temperature stress. The treatment methods were the same as those in the Figure 1 notes.

Compared with the NT, HT accelerated the seed development in thickness at the early stage of grain filling (11 days after pollination (DAP)), but significantly decreased the seed thickness at 35 DAP (Figure 2A, Table S1). The Spd pretreatment significantly increased seed thickness at 35 DAP under high temperature, while the CHA decreased the seed thickness. However, the negative impact of CHA treatment on seed thickness could be reversed by additional Spd treatment (Figure 2A). In addition, there was no significance difference between NT and HT on seed length and width (data not shown).

We also measured the 1000-grain weight of seeds of different treatment at each harvest time. At 11 DAP, HT-accelerated seeds gained weight compared with seeds under NT. At 16 DAP, 28 DAP, and 35 DAP, HT showed lower seed weight than the respective NT seeds, which could be ameliorated by Spd treatment but worsened by CHA treatment (Figure 2B).

Since the trends of variation of rice seeds harvested at 28 DAP and 35 DAP were the same, we focused on rice seeds 35 DAP in the following experiments.

### 2.2. Exogenous Spd Could Increase Rice Seed Germination and Seedling Growth under High Temperature Stress

At 11 DAP, HT showed significantly higher shoot length, seedling dry weight, germination percentage, germination index, and vigor index compared with NT (Table 1). However, from 16 DAP to 35 DAP, the shoot length, seedling dry weight, and vigor index of HT decreased significantly. Plants pretreated with Spd had significantly higher shoot length, seedling dry weight, and vigor index than plants in the HT, while plants in the CHA treatment had the opposite response (Table 1). The CHA + HT + Spd treatment significantly increased seed germination and seedling growth compared to single CHA treatment.

### 2.3. Exogenous Spd Could Increase the Starch Content in Rice Seed under High Temperature Stress

**Figure 3:**
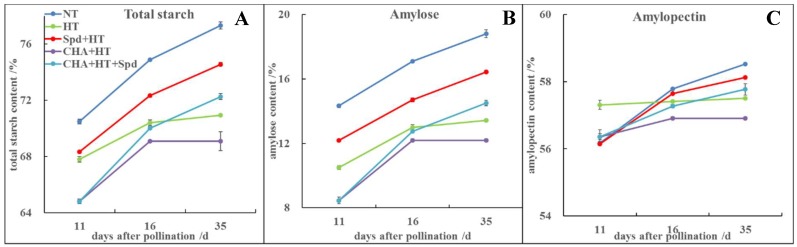
Effects of exogenous Spd and CHA on the contents of (**A**) total starch, (**B**) amylose, and (**C**) amylopectin in rice seeds at different developmental stages under high-temperature stress. The treatment methods were the same as those in the Figure 1 notes.

HT accelerated the accumulation of amylopectin at 11 DAP, but decreased the content of total starch and amylose compared with the NT during seed development (Figure 3). However, Spd pretreatment had significantly higher total starch, amylose, and amylopectin content than HT from 11 DAP to 35 DAP, whereas that of the CHA treatment showed opposite trends (Figure 3A–C). Spd treatment after CHA treatment significantly increased the accumulation of total starch, amylose, and amylopectin compared to the single CHA treatment.

**Figure 4:**
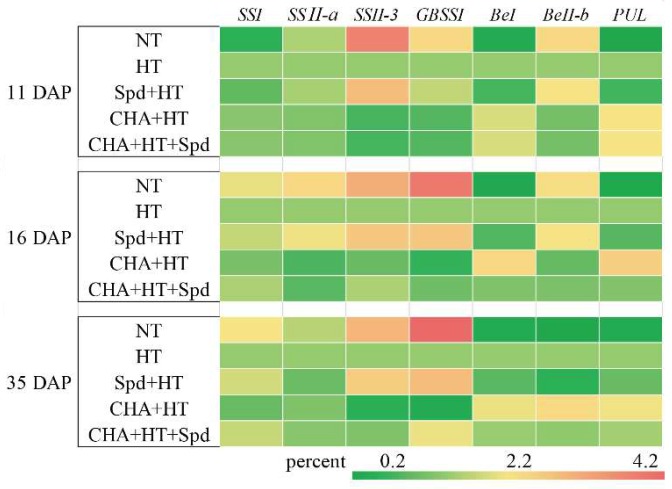
Effects of exogenous Spd and CHA on the starch synthesis-related gene expressions of rice seeds during development under high temperature (40 °C, 5 days). The expression level of HT was regarded as 1.0. The treatment methods were the same as those in the Figure 1 notes.

The expression of *GBSSI* increased gradually with the growth of the seeds in the NT treatment, and the expression of *GBSSI* obviously decreased the HT control. While the expression level of *GBSS1* in the Spd treatment was slightly lower than that in NT, it showed an obvious upward trend that was consistent with the normal temperature control (Figure 4). The expression of *SS I* and *SS II-3*, but not *SS II-a*, was also slightly higher in Spd-treated seeds than in the seeds in the other treatments (Figure 4). The expression of the starch debranching enzyme (DBE) pullulanase (*PUL*) was inhibited by high temperature stress, while Spd could reduce this effect of stress (Figure 4). *BEI* and *BEII-b* had similar patterns of response to high temperature. Both were decreased by high-temperature stress but partly increased by Spd treatment (Figure 4). CHA treatment had the opposite effect to the Spd treatment for all of the abovementioned genes (Figure 4).

### 2.4. Exogenous Spd Treatment Had a Positive Effect on the Levels of Endogenous Spd and Spm under High-Temperature Stress

**Figure 5:**
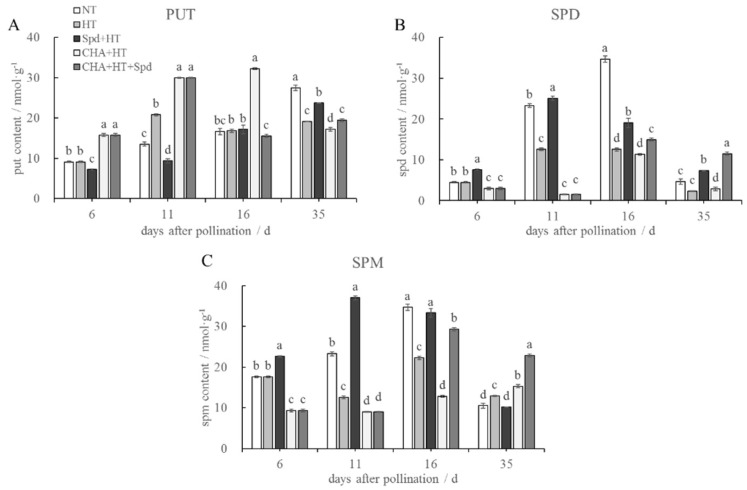
Effects of exogenous Spd and CHA on endogenous (**A**) Put, (**B**) Spd, and (**C**) Spm content of rice seeds at different developmental stages under high temperature (40 °C, 5 days). The bars mean standard deviation and different letters indicate significant differences between treatments at the same developmental stage (α = 0.05, LSD). The treatment methods were the same as those in the Figure 1 notes.

At 6 DAP, before treatment with high temperature, Spd treatment resulted in an increase in endogenous Spd and Spm content but a decrease in their precursor (Put). The opposite trend was observed in the CHA treatment (Figure 5A−C). After 5 days of high-temperature treatment (11 DAP), the content of Spd and Spm decreased significantly, but the Put content increased (Figure 5). These changes could be reversed by Spd treatment and exaggerated by CHA treatment (Figure 5). At 16 DAP, seeds of the Spd treatment showed significantly higher Spd and Spm content than those of the HT treatment. At 35 DAP, Spd treatment showed significantly higher Spd but lower Spm than that of HT.

**Figure 6:**
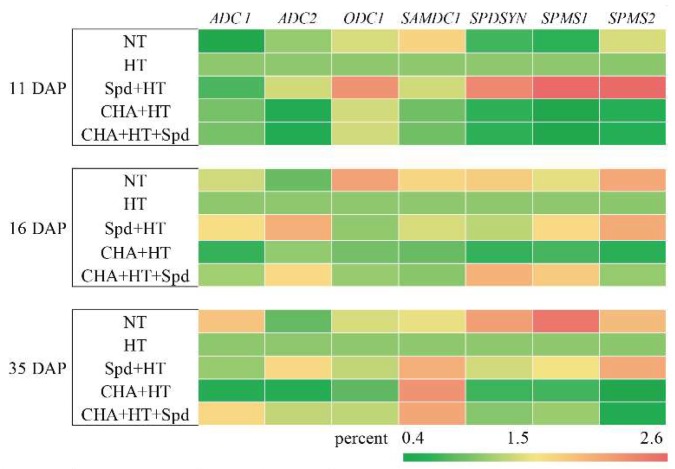
Effects of exogenous Spd and CHA on polyamine synthesis-related gene expressions of rice seeds during development under high temperature (40 °C, 5 days). The expression level of HT was regarded as 1.0. The treatment methods were the same as those in the Figure 1 notes.

To elucidate the molecular basis for changes in the polyamine biosynthesis in response to high temperature stress, the expression levels of gene-encoding enzymes involved in polyamine biosynthesis were analyzed. The polyamine synthesis-related genes include *arginine decarboxylase* (*ADC*), *ornithine decarboxylase* (*ODC*), *S-adenosylmethionine decarboxylase* (*SAMDC*), *spermidine synthase* (*SPDSYN*), and *spermine synthase* (*SPMS*). Under high temperature, the expression of polyamine synthesis-related genes was generally up-regulated by Spd treatment and down-regulated by the CHA treatment. Among these genes, *SPDSYN*, *SPMS1,* and *SPMS2* were more obviously increased by Spd treatment but decreased by the CHA treatment (Figure 6). The positive impact of Spd treatment on the expression of genes involved in polyamine biosynthesis most likely contributed to the increased content in Spd and Spm in seeds after Spd treatment. *ODC* expression was inhibited by high-temperature stress, and Spd treatment obviously increased the expression of *ODC* (11 DAP and 35 DAP, Figure 6). In addition, our treatment caused less obvious changes in the expression of *ADC1*, *ADC2*, and *SAMDC* than that of other genes.

### 2.5. Exogenous Spd Could Promote the peroxidase (POD) Activity and Decrease MDA Content under High Temperature Stress

**Figure 7:**
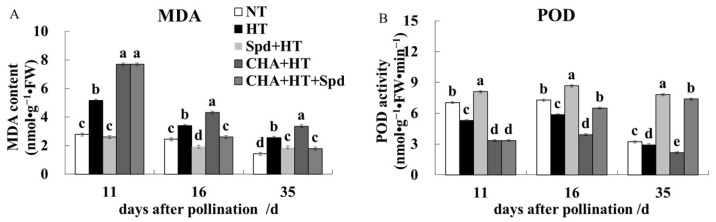
Effects of exogenous Spd and CHA on (**A**) the malondialdehyde (MDA) content and (**B**) antioxidant enzyme peroxidase (POD) activity of rice seeds during development under high temperature (40 °C, 5 days). The bars mean standard deviation and different letters indicate significant differences between treatments at the same developmental stage (α = 0.05, LSD). The treatment methods were the same as those in the Figure 1 notes.

In comparison with the NT treatment, HT caused an increase of MDA content and a decrease of POD activity (Figure 7), which could be ameliorated by Spd treatment but exaggerated by CHA treatment. The effect of CHA treatment could also be reversed by the additional application of Spd (Figure 7). 

## 3. Discussion

High temperature occurring at the early grain filling stage induced a high ratio of chalky seeds and accelerated seed maturation; it also had a significantly negative effect on seed size, weight, and seed vigor with seed maturity. Our findings are similar to those reported by Liu et al. [25], who found that high temperature formed in the sealed plastic greenhouse significantly decreased amylose, total starch content, and grain yield, while it increased the percentage of chalky seeds and relative content of amino acids and protein in conventional rice grains. The formation of grain chalkiness was considered a result of imperfectly filled starch granules under high temperature [26]. The shape and size of the grain depended on the size and quantity of the endosperm cells [27]. Morita et al. showed that 34 °C at night decreased the grain growth rate at the early or middle stages of grain filling, and reduced the seed width and thickness by inducing a reduction of cell size midway between the central point and the surface of the endosperm [27]. However, in our study, there was no significant effect on seed length and seed width, although the seed thickness reduced significantly after high temperature treatment (Figure 2A), which might have been caused by the application of different high temperature treatments at different maturing stages between the studies.

In this study, we found that exogenous Spd pretreatment improved the seed size, weight, and seed vigor, which significantly ameliorated the negative impacts of high-temperature stress on rice seed quality during early grouting period. In addition, the negative effect of CHA could be partially mitigated by an additional application of Spd. This was similar to Tang et al. (2018) [28], who found that spraying leaves with 1 mM Spd after flowering enhanced the grain-filling rate, 1000-grain rate, and grain yield in a 37.5 °C stress treatment during the early grain-filling period in conventional rice. Furthermore, Muhammad et al. [29] found that Spd priming could significantly improve seed germination and enhance seed vigor, which was similar to our results (Table 1). The significant increase of seed quality might be explained from the following three aspects: Antioxidant system, polyamine metabolism, and starch metabolism.

POD activity was positively correlated with the seed vigor index, while MDA content was negatively correlated with the seed vigor index [30], which could be used as the candidate indexes for seed vigor evaluation. It was proposed that Spd could protect biofilms and macromolecules and maintain organelle integrity under stress conditions [31]. Spd application decreased MDA content under salt stress in rice seedlings [32]. Exogenous Spd could decrease MDA content, scavenge ROS in tomato cells, and participate in the response of antioxidant enzyme systems under stress conditions, mainly increasing the activity of SOD (superoxide dismutase), POD, and CAT (catalase) [31]. Tang et al. (2018) found that leaves sprayed with 1 mM Spd after flowering showed an increase in SOD and POD activity, and decreased MDA content in conventional rice [28]. Similarly, in our study, MDA content was always lower and POD activity was always increased in Spd treated rice compared with that in the high temperature control, whereas MDA content and POD activity in the CHA treatment was always the opposite (Figure 7). 

At the early stage of seed germination, the content of endogenous polyamines changed, and this was often accompanied by or was prior to the synthesis of nucleic acids and proteins [33]. PAs induced RNA and protein synthesis in seeds [34,35], indicating that PAs played an important role in promoting seed vigor. Cao et al. (2010) found that Spd might be closely related with the comprehensive physiological changes in sweet corn seeds during development, while the Spm concentration might be used to forecast seed germination ability [36], which was similar to our results. Sagor et al. (2013) revealed that the Spm content and the survival rate in overexpressing *SPMS* Arabidopsis were much higher than those of the Arabidopsis *spms* mutant, indicating that Spm content was positively related to thermotolerance [16]. Duan et al. (2008) found that 0.1 mM Spd application markedly inhibited the accumulation of free Put but promoted the content of free Spd and Spm under salt stress conditions [37], which might be a result of the transcriptional regulation of the polyamine biosynthetic genes (*ADC, SAMDC,* and *SPDS*) [38]. Kasukabe et al. found that *FSPDS* transgenic Arabidopsis exhibited a significant increase in *SPDS* activity and Spd content in leaves, together with enhanced tolerance to various stressors including chilling, freezing, salinity, hyperosmosis, drought, and paraquat toxicity [39]. Similar results were also reported in sweet potato [40]. Therefore, Spd and Spm were suggested to be more closely related to stress resistance than Put, which was consistent with our results (Figure 5). In the present study, the application of exogenous Spd obviously increased the accumulation of endogenous Spd and Spm, which might be partly attributable to the import of spermidine. On the other hand, the enhanced expressions of *SPDSYN*, *SPMS1,* and *SPMS2* might also indirectly contribute to the synthesis of Spd and Spm (Figure 5 and Figure 6), and the improved thermotolerance of rice seeds.

**Figure 8:**
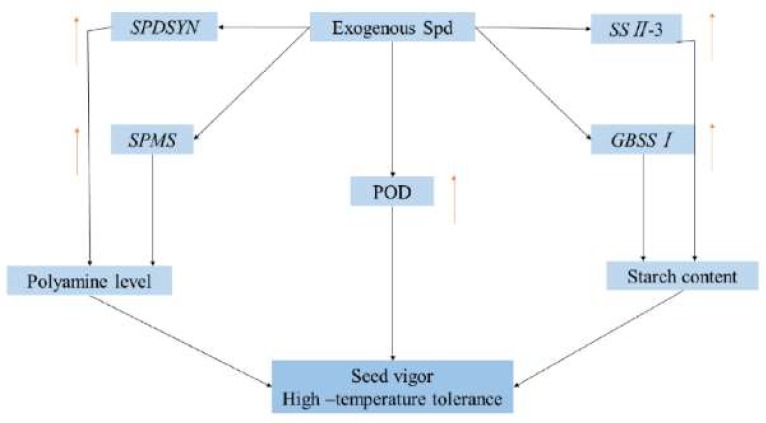
Schematic presentation of main metabolic pathways regulated by Spd in rice filling exposed to high-temperature stress.

Studies have also shown that Spd is involved in starch metabolism [41,42]. Yang et al. found that Spd application on spikes significantly increased starch content in wheat at the post-anthesis stage under water deficit [41]. In the present study, exogenous Spd significantly increased starch accumulation under high-temperature stress, while CHA treatment decreased starch accumulation (Figure 3). Zhang et al. found that the amylose and amylopectin content had greater impacts on seed vigor than total starch, while hybrid rice seeds with higher vigor generally contained higher amylose and lower amylopectin [43]. In addition, the insufficient accumulation of endosperm starch easily caused low activity of sweet corn seeds [44]. This was similar to our findings that the seeds in the Spd treatment showed higher seed vigor with higher amylose content under high temperature (Figure 3, Table 1). Amylose content is an important factor that affects the cooking and eating quality of rice, and was greatly affected by environmental conditions [45]. Wang et al. (2012) found Spd significantly increased the activities of SSSase, while MGBG showed an opposite result, indicating the possible effect of Spd on starch synthase [42]. In our study, Spd mainly increased the expressions of *SSII-3* and *GBSSI* (Figure 4) and enhanced the amylose and total starch accumulation (Figure 3B), which might be the reason for higher rice seed quality under high-temperature stress induced by Spd. Hence, Spd is likely involved in the establishment of high-temperature tolerance during rice grain filling (Figure 8).

## 4. Materials and Methods

### 4.1. Plant Material and Cultivation Conditions

The plants of hybrid rice (*Oryza sativa* L. ssp. *indica*) “YLY 689” were grown in a greenhouse (60% relative humidity) with a 16-h light (30 °C)/8-h dark (20 °C) photoperiod (normal conditions).

At 3 DAP, the plants were sprayed with 4 mL 1.5mM Spd or 20 mM CHA per spikelet once a day for three days (from 3 DAP to 5 DAP). Plants treated with distilled water were used as the control.

Then, plants were transferred into high-temperature growth chambers (60% relative humidity) with a 16-h light (40 °C)/8-h dark (30 °C) photoperiod for 5 days (from 7 DAP to 11 DAP, HT, Spd + HT, CHA + HT). Plants under normal conditions (60% relative humidity) with a 16-h light (30 °C)/8-h dark (20 °C) photoperiod were used as control (NT). At 11 DAP, all plants were transferred to normal conditions with a 16-h light (30 °C)/8-h dark (20 °C) photoperiod.

Moreover, some plants treated with CHA were additionally sprayed with 1.5 mM Spd for three days (from 12 DAP to 15 DAP) (CHA + HT + Spd). To study the effect of Spd treatment on mature seed quality, all rice plants were harvested until 35 DAP. Sampling time settings in this study are shown in Table 2. Twenty spikelets were randomly selected at each sampling time for each treatment, and the middle grains were taken for testing.

### 4.2. Measurement of Morphological Index of Rice Grain

Each sample had three replications, and each replicate contained 50 seeds. The seed length, width, and thickness were measured by Vernier calipers. The 1000-grain weight of each sample was tested on a balance. Grain quality (chalky or normal) was observed following the standard for classification of rice grains. ‘Normal’ grains that exhibited a transparency and ‘chalky’ grains that contained opaque part(s) within the endosperm were counted from these harvested grains according to the National Standard of the People’s Republic of China ‘GB t17891-1999 high quality rice.’

### 4.3. Germination Test

The seeds of different treatments sampled at each harvest time (Table 1) were used for the seed standard germination test. The seeds were placed on moistened germinating paper and each sample was replicated three times (100 seeds for each replicate). Seeds were then incubated in a growth chamber with a 16-h light (30 °C)/8-h darkness (20 °C) photoperiod for 14 days. Germinated seeds (seed radicle visibly protruded through the seed coat and reached half of the length of the whole seed) were counted every day. The germination energy (GE) and germination percentage (GP) were calculated at the 5th and 14th day, respectively. After 14 days of germination, root length (RL) and shoot height (SH) were measured, and the seedling dry weight (SDW) was determined after drying at 80 °C for 24 h [46]. The above measurements were based on ten randomly selected normal seedlings for each replication. In addition, the germination index (GI) was calculated according to GI = Σ (Gt/Dt), where Gt is the number of germinated seeds per day, t is the time corresponding to Gt in days, and Dt is the number of germination days. Seed vigor index (VI) was determined according to the formula VI = GI × seedling dry weight [46].

### 4.4. Measurement of Physiological Parameters

Malondialdehyde (MDA) concentration was determined using the thiobarbital (TBA) reaction as described by Zhu et al. [47] with some modifications. Crude enzyme solution (1.50 mL) was added to 2.50 mL of TBA–TCA solution, boiled in water for 15 min, rapidly cooled to room temperature, centrifuged at 1800 r·min^−1^ for 10 min, and the supernatant was removed. The absorbance of samples was measured at 532 and 600 nm. MDA content (nmol·g^−1^) was calculated as [(D532 − D600) × A × V/a]/(1.55 × 10^−1^ × w), where A is the total amount of the reaction solution, V is the total amount of the extract, a is the extract for the measurement, w is the fresh weight of the sample, and 1.55 × 10^−1^ is the extinction coefficient of MDA.

Peroxidase (POD) activity was tested as described by Zhu et al. [47] with some modifications. The assay mixture consisted of a 1.35 mL 25 mM phosphate buffer (pH 7.0), 100 μL 1.5% guaiacol, 100 μL 100 mM H_2_O_2_, and 100 μL enzyme extract. The increase in absorbance caused by oxidation of guaiacol was measured at 470 nm for 2 min. The enzymatic activity was calculated as nmol of guaiacol oxidized min^−1^·g^−1^ FW at 37  °C, and was expressed as nmol·g^−1^·FW·min^−1^. Three replications for each sample were conducted. 

### 4.5. Measurement of Starch Content

Amylose, amylopectin, and total starch in rice seeds were determined by dual-wavelength spectrophotometer according to the report by Zhou et al. [48] with some changes. The pulverized dry sample was weighed, degreased with anhydrous ether, and the ether was removed. The degreased sample was weighed to 0.1 g, placed in a 50.00 mL volumetric flask. 10 mL of 0.5 mol/L KOH solution was added to the sample. The sample was then dispersed and dissolved in a water bath at 75 °C for 10 min, and diluted with water to the mark. The mixture was let stand for 15 min. Then the filtrate was distilled to 2.50 mL, put in a 50.00 mL volumetric flask, 0.5 mL of iodine reagent was added, and diluted to the mark with water. The mixture was let stand for 15 min. The absorbance value of the sample solution was measured. The standards (Sigma) for amylose and amylopectin were used for the production of standard curves. Starch analyses were performed with three replications.

### 4.6. Measurement of Endogenous Polyamine Content

The extraction and HPLC analysis of PAs was conducted as described by Huang et al. [15]. Seeds (0.3 g) from the different treatments at each harvest time were homogenized with 2 mL of 5% (*w/v*) cold perchloric acid. The mixtures were kept in an ice bath for 1 h and then centrifuged at 10,000 ×  *g* for 30 min at 4 °C. The supernatant was transferred and stored at −80 °C for quantification of PAs. Five hundred microliters of the above solution were collected, and 1 mL 2 mol·L^−1^ NaOH solution and 10 μL benzoyl chloride were added to the solution. The mixtures were incubated for 20 min at 37 °C. Two milliliters of saturated NaCl solution and 2 mL of diethyl ether were added into the above mixture, then it was centrifuged at 1500 ×  *g* for 15 min at 4 °C. One milliliter of diethyl ether phase was extracted, dried by nitrogen, and redissolved in 100 μL of methanol for the following test. The extracts above were filtered through a 0.22 μm membrane filter, and then eluted at room temperature through a 6.0 mm × 150 mm, 5 mm particle size reverse-phase (C18) column (Shim-Pack CLC-ODS). Peaks of PAs were detected by an SPD-20A (Shimadzu) absorbance detector at 254 nm. The mobile phases consisted of water and methanol (35/65, *v*/*v*) at a flow rate of 1.0 mL·min^−1^. The polyamine standards (Sigma) of Put, Spd, and Spm were used for the production of standard curves. Analysis of PAs was performed with three replications.

### 4.7. Real-time Fluorescence Quantitative PCR

Total RNA of each sample of different treatments at each harvest time was extracted and 500 ng of RNA was reverse-transcribed using PrimeScript^TM^ RT reagent Kit (Takara, Dalian, China). Real-time fluorescence quantitative polymerase chain reaction (RT-PCR) was carried out using CFX96TMReal Time PCR Detection System (Bio-Rad, Hercules, CA, USA). Primer sets were designed with the Primer5 software (Table 3), and the rice β-Actin gene (LOC_Os03g50890) was used as an internal control. The reaction system consisted of a total of 20 μL containing 1 μL diluted cDNA, 0.6 μL forward and reverse primers, 7.8 μL ddH_2_O, and 10 μL AceQ qPCR SYBR Green Master Mix (Vazyme, Nanjing, China). The abundance of transcribed genes was calculated using the relative 2-^ΔΔ^CT analytical method. Three biological replicates were used and each biological replicate was technically repeated three times. All data were expressed as the mean SD (standard error of the mean) after normalization.

### 4.8. Statistical Analysis

The data were subjected to an analysis of variance (ANOVA) on the Statistical Analysis System (SAS) software. The multiple comparisons for mean values were performed by the least significant difference (LSD) test (α = 0.05). Before ANOVA, the data were transformed according to y  =  arcsin [sqrt (x/100)].

## Figures and Tables

**Table 1:** Effects of exogenous Spd and CHA on seed germination and seedling growth during rice seed maturity after high-temperature stress (40 °C, 5 days).

Treatment	DAP	SH (cm)	SDW (g)	GP	GI	VI
NT	11	5.04 ± 0.03b	0.028 ± 0.001b	0.42 ± 0.012b	2.63 ± 0.27b	0.07 ± 0.007c
HT		5.91 ± 0.05a	0.036 ± 0.001a	0.50 ± 0.012a	3.40 ± 0.17a	0.12 ± 0.007a
Spd + HT		4.43 ± 0.05c	0.032 ± 0.001b	0.42 ± 0.012a	2.91 ± 0.03ab	0.09 ± 0.002b
CHA + HT		2.83 ± 0.04d	0.019 ± 0.001c	0.34 ± 0.010c	1.75 ± 0.15c	0.03 ± 0.003d
CHA + HT + Spd		2.83 ± 0.04d	0.019 ± 0.001c	0.34 ± 0.010c	1.82 ± 0.11c	0.03 ± 0.003d
NT	16	8.12 ± 0.06a	0.053 ± 0.001a	0.67 ± 0.093ab	4.52 ± 0.58a	0.24 ± 0.028a
HT		5.70 ± 0.08d	0.040 ± 0.001c	0.62 ± 0.012b	3.64 ± 0.06bc	0.15 ± 0.002b
Spd + HT		7.25 ± 0.06b	0.049 ± 0.001b	0.76 ± 0.012a	4.21 ± 0.05ab	0.21 ± 0.003a
CHA + HT		4.57 ± 0.06e	0.031 ± 0.001e	0.55 ± 0.017b	2.54 ± 0.16d	0.08 ± 0.004c
CHA + HT + Spd		6.60 ± 0.15c	0.046 ± 0.001d	0.65 ± 0.007ab	3.27 ± 0.05cd	0.15 ± 0.004b
NT	35	8.89 ± 0.08a	0.100 ± 0.001a	0.85 ± 0.018a	6.45 ± 0.32a	0.64 ± 0.031a
HT		5.90 ± 0.01c	0.052 ± 0.002c	0.59 ± 0.007c	3.82 ± 0.09c	0.20 ± 0.011c
Spd + HT		7.93 ± 0.04b	0.063 ± 0.001b	0.87 ± 0.007a	6.06 ± 0.12a	0.38 ± 0.006b
CHA + HT		5.48 ± 0.33c	0.044 ± 0.001d	0.43 ± 0.007d	2.48 ± 0.15d	0.11 ± 0.008d
CHA + HT + Spd		7.72 ± 0.10b	0.054 ± 0.001c	0.63 ± 0.013b	3.85 ± 0.10b	0.21 ± 0.006c

NT: Normal temperature control; HT: High temperature control; Spd + HT: 1.5 mM exogenous Spd treatment + high temperature; CHA + HT: 20 mM exogenous CHA treatment + high temperature; CHA + HT + Spd: 20 mM exogenous CHA treatment + high temperature + exogenous 1.5 mM Spd; SH, shoot height; SDW, seedling dry weight; GP, germination percentage; GI, germination index; VI, vigor index; DAP: Days after pollination. The data were presented as the mean ± standard deviation values and different letters indicated significant differences between treatments (α = 0.05, least significant difference (LSD)).

**Table 2:** Sampling time points of different treatments during development of rice seed.

	DAP	3–5 (Spd or CHA Sprayed)	6	7–11 (High Temperature Treatment)	11	12–15 (Spd Sprayed Based on CHA + HT)	16	28	35
Treatment									
NT		--	√	--	√	--	√	√	√
HT		--	--	--	√	--	√	√	√
Spd + HT		--	√	--	√	--	√	√	√
CHA + HT		--	√	--	√	--	√	√	√
CHA + HT + Spd		--	--	--	--	--	√	√	√

DAP: Day after pollination; NT: Normal temperature control with a 16-h light (30 °C)/8-h dark (20 °C) photoperiod; HT: High temperature treatment with a 16-h light (40 °C)/8-h dark (30 °C) photoperiod for 5 days (from 7 DAP to 11 DAP); Spd + HT: 1.5 mM exogenous Spd treatment + high temperature; CHA + HT: 20 mM exogenous CHA treatment + high temperature; CHA + HT + Spd: 20 mM exogenous CHA treatment + high temperature + exogenous 1.5 mM Spd; All spray treatments were done once a day for three days; “√” indicated the sampling day of different treatments during development of rice seed. “--” indicated no sampling.

**Table 3:** Primer sequences used in RT-PCR (Real-time fluorescence quantitative polymerase chain reaction).

Locus ID	Gene		Primer Sequence
LOC_Os06g04070	*ADC1*	FW	CGTCATCGACGTTGGTGGA
		RW	CCAAGCTGTATGCCACGGAC
LOC_Os04g01690	*ADC2*	FW	AGAAGGTTGCGACGGAGAATG
		RW	TGGTCAGCCCTTTCTTCATCA
LOC_Os09g37120	*ODC1*	FW	CGGCTGGCTCCAACTTCAA
		RW	TGGAGTATGCCAGGTGGATCTT
LOC_Os04g42095	*SAMDC1*	FW	GTCTTTGCTGACCCTGATGG
		RW	CGTGCAAGATCCAGAACAGAG
LOC_Os07g22600	*SPDSYN*	FW	GGTGTTTCAGTCCTCCACGTA
		RW	TCCCTCTCAGTGACCTGAATC
LOC_Os06g33710	*SPMS1*	FW	CCTGGTGGAGTTCTATGC
		RW	CACTGCTGGACCTTCTTT
LOC Os02g15550	*SPMS2*	FW	AGAGCATGTGGTTGCATACGC
		RW	AACCCTTGAATGTCTCACGGC
LOC_Os06g04200	*GBSSI*	FW	ACCTGACACTGGAGTTGATTAC
		RW	GTATGGGTTGTTGTTGAGGTTTAG
LOC_Os06g06560	*SSI*	FW	GTCTTGTGCCAGTCCTTCTT
		RW	CACACCCTGATGTGCTAGATTAT
LOC_Os02g51070	*SSII-2*	FW	CAGGGCCAAATGTGATGAATG
		RW	CTCTTCTTGCCAGAGCCTTAG
LOC_Os06g12450	*SSII-3*	FW	CTGCACTCCTGCCTGTTTAT
		RW	GCCCTGGTAAGCGATATTATGT
LOC_Os06g51084	*Be*	FW	AATGGGCATGCATCGACATC
		RW	CTGGTTCTTGCCCTTCCCTA
LOC_Os02g0528200	*BEIIb*	FW	GGCATGCTAGAGTTTGACCG
		RW	TCCACCAAAGAGTCCAGCAT
LOC_Os04g0164900	*PUL*	FW	GGTCTGTTCTTGGAGCCTAAT
		RW	CAGCTAGTCCGATCTGTATGTG

## Supplementary Materials

The supplementary materials are available online.
