# Supplementary material for: Spermidine Enhances Heat Tolerance of Rice Seeds by Modulating Endogenous Starch and Polyamine Metabolism
Source: Molecules. 2019 Apr 9;24(7):1395. doi: 10.3390/molecules24071395 (PMC6480098; doi:10.3390/molecules24071395)
Supplement: Supplementary file 1 [file molecules-24-01395-s001.pdf]

Table S1. Effects of exogenous Spd and CHA on the rice seed size at different developmental stages under high-temperature stress.

| Treatment | Days after pollination | Length/mm | Width/mm |
|---|---|---|---|
| NT | | 8.34±0.100a | 2.40±0.044a |
| HT | | 8.33±0.108a | 2.53±0.095a |
| HT+SPD | 11 | 8.31±0.309a | 2.49±0.041a |
| HT+CHA | | 8.39±0.110a | 2.48±0.027a |
| HT+CHA+Spd | | 8.39±0.110a | 2.48±0.027a |
| NT | | 8.34±0.134a | 2.42±0.043a |
| HT | | 8.37±0.056a | 2.46±0.038a |
| HT+SPD | 16 | 8.39±0.081a | 2.52±0.057a |
| HT+CHA | | 8.39±0.058a | 2.48±0.050a |
| HT+CHA+Spd | | 8.39±0.056a | 2.43±0.038a |
| NT | | 8.34±0.100a | 2.20±0.051a |
| HT | | 8.37±0.087a | 2.18±0.058b |
| HT+SPD | 28 | 8.39±0.080a | 2.29±0.046a |
| HT+CHA | | 8.39±0.042b | 2.08±0.053b |
| HT+CHA+Spd | | 8.39±0.141a | 2.08±0.043a |
| NT | | 8.34±0.131a | 2.22±0.052a |
| HT | | 8.37±0.072a | 2.10±0.034ab |
| HT+SPD | 35 | 8.39±0.131a | 2.24±0.055a |
| HT+CHA | | 8.39±0.120b | 2.02±0.046ab |
| HT+CHA+Spd | | 8.39±0.137a | 2.07±0.061b |

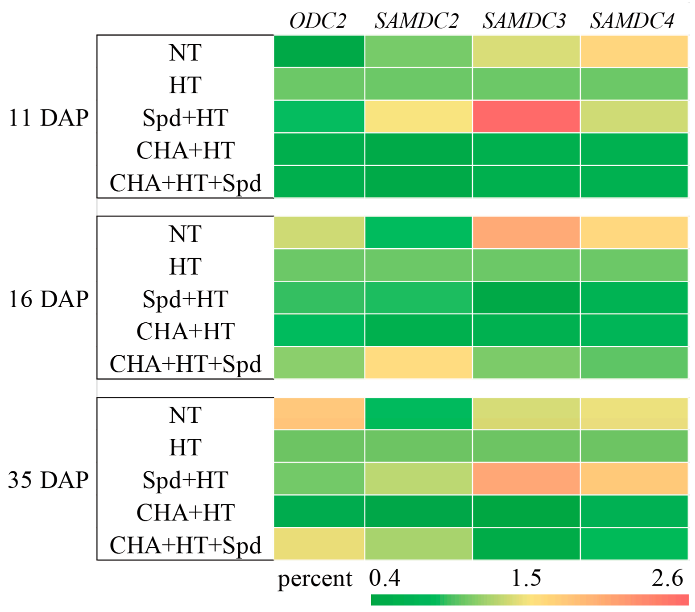


Figure S1. Effects of exogenous Spd and CHA on polyamine synthesis-related genes (putative *ODC2, SAMDC2, SAMDC3, SAMDC4*) expressions of rice seeds during development under high-temperature (40°C, 5 days). The expression level of HT was regarded as 1.0.

NT: Normal temperature control; HT: High temperature control; Spd+HT: 1.5mM exogenous Spd treatment + high temperature; CHA+HT: 20mM exogenous CHA treatment + high temperature; CHA+HT+Spd: 20mM Exogenous CHA treatment + high temperature + exogenous 1.5mM Spd.
